# *Lactococcus garvieae*: a small bacteria and a big data world

**DOI:** 10.1186/2047-2501-3-S1-S5

**Published:** 2015-02-24

**Authors:** Guillermo López-Campos, Mónica Aguado-Urda, María Mar Blanco, Alicia Gibello, María Teresa Cutuli, Victoria López-Alonso, Fernando Martín-Sánchez, José F Fernández-Garayzábal

**Affiliations:** 1 Health and Biomedical Informatics Centre (HABIC), The University of Melbourne, Melbourne, Victoria, 3010, Australia; 2 Faculty of Veterinary Sciences, Department of Animal Health, Complutense University, Madrid, 28040, Spain; 3 Computational Biology Unit, National Institute of Health "Carlos III", Madrid, 28220, Spain

**Keywords:** *Lactococcus garvieae*, Big Data, Genomics, Gene expression

## Abstract

**Objective:**

To describe the importance of bioinformatics tools to analyze the big data yielded from new "omics" generation-methods, with the aim of unraveling the biology of the pathogen bacteria *Lactococcus garvieae*.

**Methods:**

The paper provides the vision of the large volume of data generated from genome sequences, gene expression profiles by microarrays and other experimental methods that require biomedical informatics methods for management and analysis.

**Results:**

The use of biomedical informatics methods improves the analysis of big data in order to obtain a comprehensive characterization and understanding of the biology of pathogenic organisms, such as *L. garvieae*.

**Conclusions:**

The "Big Data" concepts of high volume, veracity and variety are nowadays part of the research in microbiology associated with the use of multiple methods in the "omic" era. The use of biomedical informatics methods is a requisite necessary to improve the analysis of these data.

## Introduction

*Lactococcus garvieae *is a Gram-positive bacterium able to grow in a wide range of environmental conditions (temperature, pH and salinity) making it a ubiquitous microorganism. *L. garvieae *is an important fish pathogen causing high mortality and economic loses in fishery industry [1]. Despite its major relevance as a fish pathogen, this organism that can be found as well in cattle and dairy products where it has been associated with mammal infections [2-5]. In the last couple of decades an increasing number of human infections, mostly associated with endocarditis [6-10], have raised the awareness of the importance of *L. garvieae *as an emerging potentially zoonotic pathogen and has fostered the investigation of this pathogen but despite these efforts, the genomic information available about this organism has been scarce.

The advances in molecular biology have strongly influenced all areas in biological research including microbiology. These advances and the development of new analytical techniques have increased the capability of these laboratories to generate new data by several orders of magnitude. As a consequence of this data explosion in the last couple of decades all biological sciences, including microbiology, have increasingly become information intensive sciences. In this regards the development twenty years ago of the first microarray based technologies [11] opened the doors for the first real "-omics" data gathering applications and fostered the generation of massive amounts of data coming from the simultaneous screening of thousands of genes. For more than a decade microarrays remained as the major genomic data source in biology until a new technological breakthrough was developed in the form of the massive parallel sequencing (MPS) technologies, also called next generation sequencing [12,13]. These new technologies reduced the cost and time required for sequencing projects making them increasingly affordable. Altogether with these "-omics" data, advances have occurred as well in other areas and other techniques, such as proteomics or imaging techniques. These and other methodologies used in microbiological laboratories have nowadays transformed microbiologists into generators and users of an unprecedented amount and diversity of data. In this context microbiology laboratories are now immersed in their own "Big Data" world, where they are facing in their own way the traditional four V's used to describe Big Data (Volume, Variety, Velocity and Veracity) [14].

Current approaches for the study of poorly understood pathogens are based in combinations of these high-throughput technologies combined with some other "classical" molecular biology techniques. In this work we present the study of *L. garvieae *as an example of how the previously cited technologies have been sequentially applied depending on their availability and development to unravel the biology of a poorly understood pathogen.

## Review

**Figure 1:**
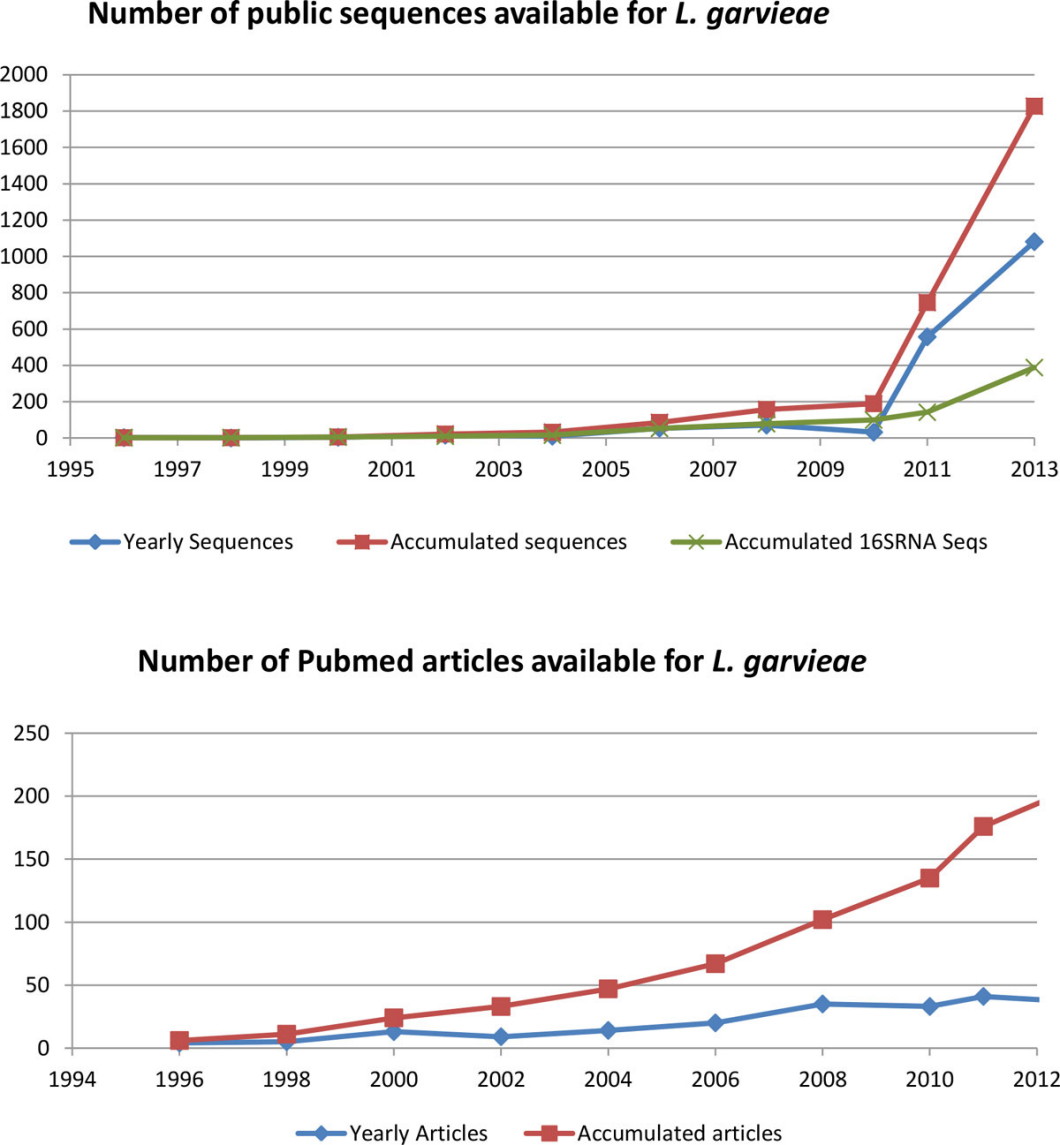
**Information available for *L. garvieae *in the main repositories of the NCBI**. The first graph shows the number of sequences available for this organisms and it is remarkable the effect of new MPS technologies in the number of sequences published. The second graph shows the steady growth in the number of references of *L. garvieae *in the literature in the recent years.

**Figure 2:**
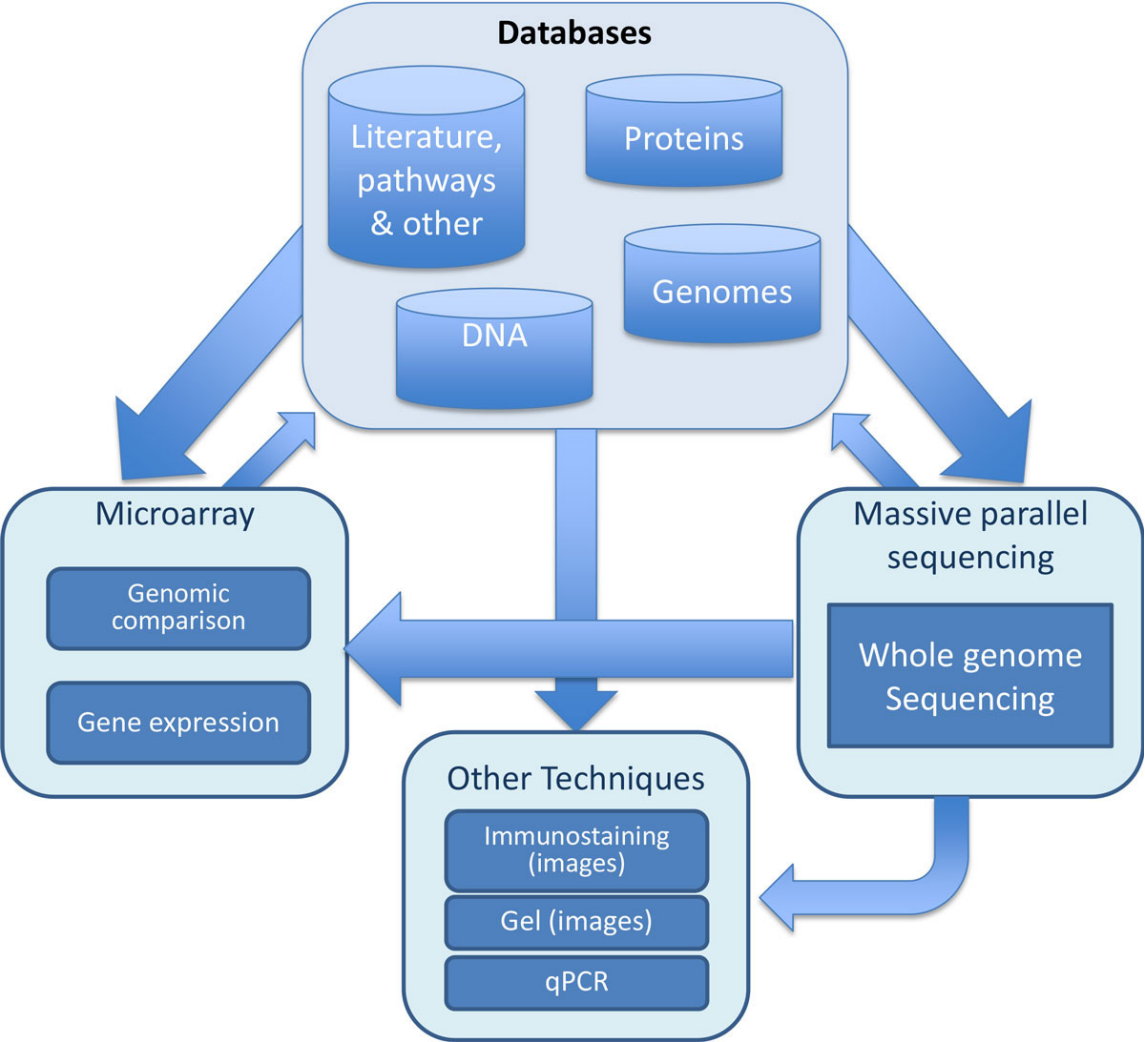
**Overview of the different methods and analyses carried out in the characterization of *L. garvieae *and the relationships among the different elements**. The arrows show the flux of data required for the design analysis or interpretation of the results between the different techniques and databases

*Lactococcus garvieae *was firstly described in 1983 [15] but the literature and molecular data associated with this organism have been scarce until very recent times (Figure 1). This paucity in the available information about this organism acted at the same time as a stimulus but also as a limiting factor in terms of the analytical methodologies that could be applied. The lack of data and absence of references also increased the complexity of genetic and genomic analyses requiring the comparison with larger datasets generated from other organisms for the interpretation of the results. As it has been pointed out previously, this work captures the evolution in the amount and variety of data generated during the genomic characterization of this increasingly important pathogen. For this purpose, a combination of different high-throughput and traditional microbiological techniques were required and applied generating a rich and diverse data environment (Figure 2).

### High throughput comparative genomics as a first approach for the genomic characterization of *L. garvieae*

As was stated previously the paucity in the availability of molecular data associated with *L. garvieae *placed a limit on the understanding of its biology and therefore the possible mechanisms associated with its pathogenicity. In a time when traditional Sanger sequencing was very time consuming and expensive, and massive parallel sequencing was still in its very early stages of development and had prohibitive costs, comparative genomic approach using microarrays was considered as feasible and affordable for the advance in the knowledge of the genomic content of *L. garvieae*. This approach was based on the comparison of the unknown genome of *L. garvieae *CECT4531 with the genomes of two whole-genome sequenced related bacteria, the non-pathogenic *Lactococcus lactis *subsp. *lactis *IL1403 and the pathogenic *Streptococcus pneumoniae *TIGR4. This comparison was carried out using gene expression microarrays designed for these reference organisms, in an experimental approach known as array based comparative genomic hybridization (aCGH) [16]. This approach exploits the fact that DNA strands can recognize and hybridize (attach) to complementary sequences (hybridization), and therefore it would make possible to identify DNA from *L. garvieae *similar enough to hybridize with the DNA from the characterized reference bacteria.

The microarrays used in these analyses consisted of a set of thousands of immobilized DNA probes designed to capture the whole set of messenger RNAs (mRNAs) of each of these organisms. Therefore, hybridizing the mostly unknown genomic DNA of *L garvieae *with the probes contained in the microarrays would provide an insight about the genes shared with these two organisms. The interpretation of the results from these experiments required the development of a bioinformatics framework that contextualized the positive calls (positive reactions, where DNA from the sample recognizes and hybridizes with the immobilized probes on the microarray) from the microarray experiments in terms of sequence similarities and provided a similarity threshold for the identified sequences.

**Figure 3:**
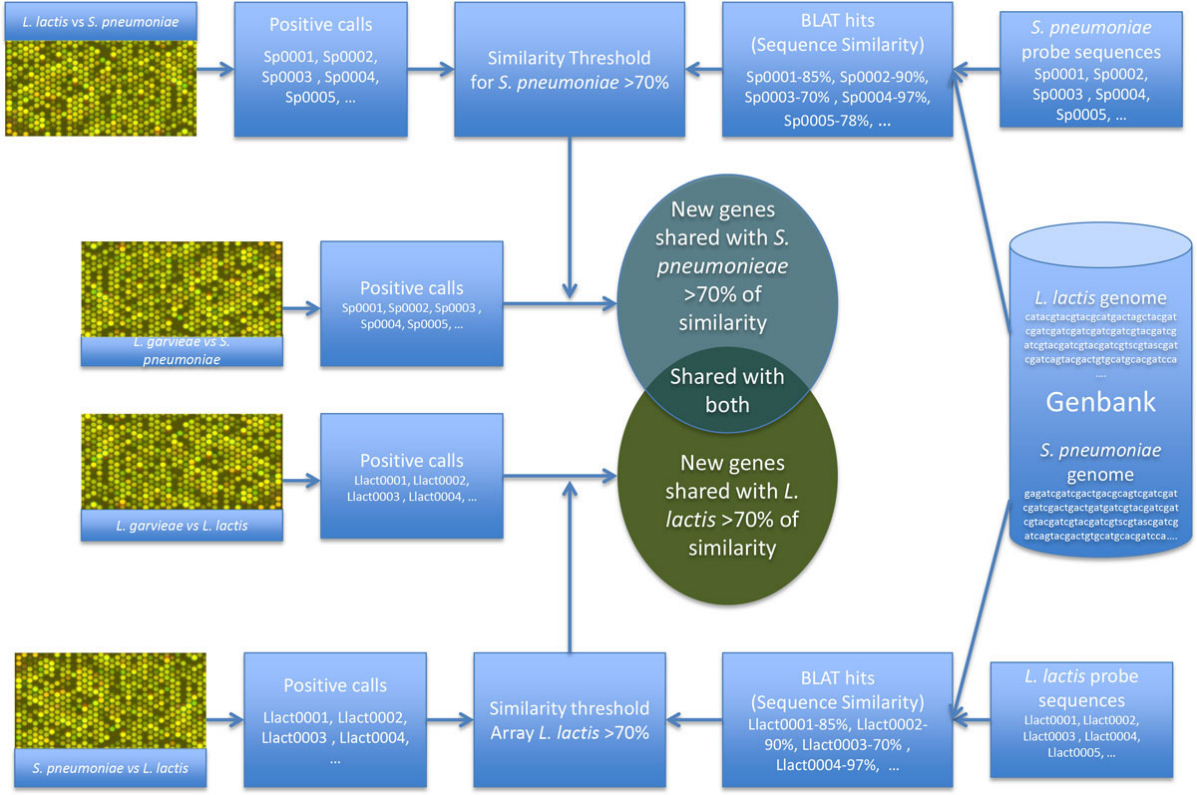
**Schema of the bioinformatics framework developed for the interpretation of the results coming from the aCGH experiments based on sequence analyses**.

The comparison framework was built combining both bench (experimental hybridizations) and "in silico" (sequence comparison) results to define the degree of similarity among the sequences identified during the aCGH experiments (Figure 3). For this purpose, a set of hybridizations between the genome of each of the reference organisms using the array of the other reference organism was carried out. These hybridizations resulted in a set of positive calls associated with genes conserved between both organisms.

Using the sequence information available for the probes on each microarray (*L. lactis *subsp. *lactis *IL1403 GEO: GPL9532 and *S. pneumoniae *TIGR4 GEO: GPL5781) and both genome sequences we carried out a sequence alignment analysis using a BLAT [17] search. This search compared the sequences of the immobilized probes with the each of the reference genomes (*L. lactis *subsp. *lactis *IL1403 Genbank: http://www.ncbi.nlm.nih.gov/nuccore/NC_002662 and *S. pneumoniae *TIGR4 Genbank: http://www.ncbi.nlm.nih.gov/nuccore/NC_003028). The results of these alignments identified probes and regions of probes that were present in the other reference genome, providing a similarity measurement between these conserved sequences.

Next step in this framework consisted in the combination of results from both experimental approaches, matching the results from hybridization with those generated from sequence comparisons. This allowed us to identify a similarity threshold, where positive calls from the hybridizations had at least a 70% of similarity (minimum similarity between the immobilized probe and the genomic sequence for positive results).

Therefore, when applied to the results from the hybridizations between *L. garvieae *and the arrays of the two reference microorganisms, this approach led us to the identification of 267 putative genes in *L. garvieae*. At the moment of the publication of these results [16] the corpus of available sequences for *L. garvieae *was limited to approximately 200 sequences, around half of them 16S RNAs. Thus, this approach based on aCGH and "in silico" analyses expanded the knowledge about the genomic content of this bacterium in an unprecedented way at that moment.

Other approaches based on comparative genomics using a different methodology called suppression subtractive hybridization combined with sequencing and further sequence characterization using BLAST programs have been used as well to identify other gene sets [18,19].

### The genomic era of *L. garvieae*

In the last decades the abundance of genomic-based techniques combined with their cost reduction have allowed a flourishing era of genomic studies in microbiology. The number of publicly available genomes and genome projects has grown enormously thanks to the maturity and reduction in costs of massive parallel sequencing (MPS) technologies. More specifically in the study of *L. garvieae*, this sequencing approach has been delayed until massive parallel sequencing (MPS) was mature enough to become a cost effective method for obtaining a "de novo" sequence of a bacterial species. The first genome sequences for *L. garvieae *were published in 2011 [20-23], followed by the sequences of another seven strains up to 2014 [24-28]. At the moment of writing there are currently 14 publicly available genome sequences for 12 different strains http://www.ncbi.nlm.nih.gov/genome/genomes/699. These sequences include three different genomes of *L. garvieae *ATCC49156 obtained using different sequencing technologies, including the version which is considered the reference genome (Genbank: http://www.ncbi.nlm.nih.gov/nuccore/NC_015930.1) [22].

From a data perspective, the final results from a MPS experiment that are distributed to the experimenters are large files containing hundreds of thousands or millions, depending on the technology, of partially overlapping reads (short DNA sequences). These reads should then be arranged in order to reconstruct longer sequences (contigs) in a process called assembly. The final aim of this assembly process is to arrange all these contigs into one single sequence that represents the original genome sequenced. Assembly is a challenging task due to the size of the sequence files, and it is affected by other factors, such as the size of the genome and the availability of a reference genome that might be used as a template for this process. In the case of *L. garvieae *there was not any reference genome close enough to be used and therefore it required the use of more complex approaches based on "de novo" assembly. This process is more complex and computationally demanding than the assembly based on a reference genome. The first three published genomes of *L. garvieae *came up almost simultaneously and they used this approach. Despite of the improvements in the quality and length of the reads coming from MPS analyses, completing a genome usually requires further analyses and filling the gaps between contigs. This process is time consuming and requires further runs of traditional sequencing, for this reason most of the genomes remain as drafts containing a variable set of contigs. In May 2014 only two of the 14 sequences available were completed. These draft genomes behave very much alike complete genomes and can be used almost equally as the complete genomes for comparative studies.

Once the genome sequence is available the next step consists in adding information to that sequence in what is called the annotation process. Annotation represents another data demanding task where firstly genes have to be identified in the genome sequence, and afterwards those potential genes have to be assigned to a particular function when possible. This process is mostly automated nowadays and usually is carried out using workflows involving different programs and databases, being the process of assigning the function the most computationally demanding. This is done generally by homology inference based on sequence alignment using programs such as BLAST [29,30], BLAST2GO [31] HMMER [30] or the RAST server [32]. Homology inference methods are more efficient and more rapidly carried out when there is available a reference strain that can be used to export the annotations. For *L. garvieae*, as it happened with the alignment stage, the lack of a close reference made this process slightly more complicated and currently not all the available sequences are fully annotated. This automated annotation process identifies regions that are predicted to be functional genes but for which no-function can be assigned and therefore they are assigned as hypothetical proteins. These cases benefit in from manually curated annotations by experts. Another important characteristic associated with many of these automated processes is that they are based on the identification of certain elements such as protein coding genes, rRNAs or tRNAs, but other important information associated with regulatory elements such as small RNAs requires specific annotation processes. For this purpose it is necessary to access to other databases, as RFAM [33] and to use different search strategies and algorithms, for example INFERNAL [34], based in many cases in the identification of certain secondary structures and models rather than in sequence similarity.

Genome annotation is associated in different ways with two Big Data concepts, "Volume" and "Veracity". "Volume" is related with annotation because during this process it is required to have access to the main sequence repositories, frequently both of proteins and nucleotides, to assign a function to the genes. On the other hand "Veracity" is a relevant concept associated with the process of annotation because it has an important impact on the final quality of the annotation. Problems could arise at different stages during the annotation process, from the gene calling (identifying genes within the genome sequence) to association of a function, leading to missing or wrongly annotated genes. Due to the fact that automatic annotation methods rely on existing information, these possible errors might be inherited and propagated during the annotation process and therefore "veracity" of the data is a concern requiring the implementation of methods for the quality control of the annotations [35].

### Validation of aCGH experiments using genome information

The bioinformatics framework developed for the analysis and the interpretation of the aCGH data was designed before any *L. garvieae *genomic data were available. The release of genomic data for 12 different *L. garvieae *strains made possible to revisit and confirm the data generated during the aCGH experiments.

**Figure 4:**
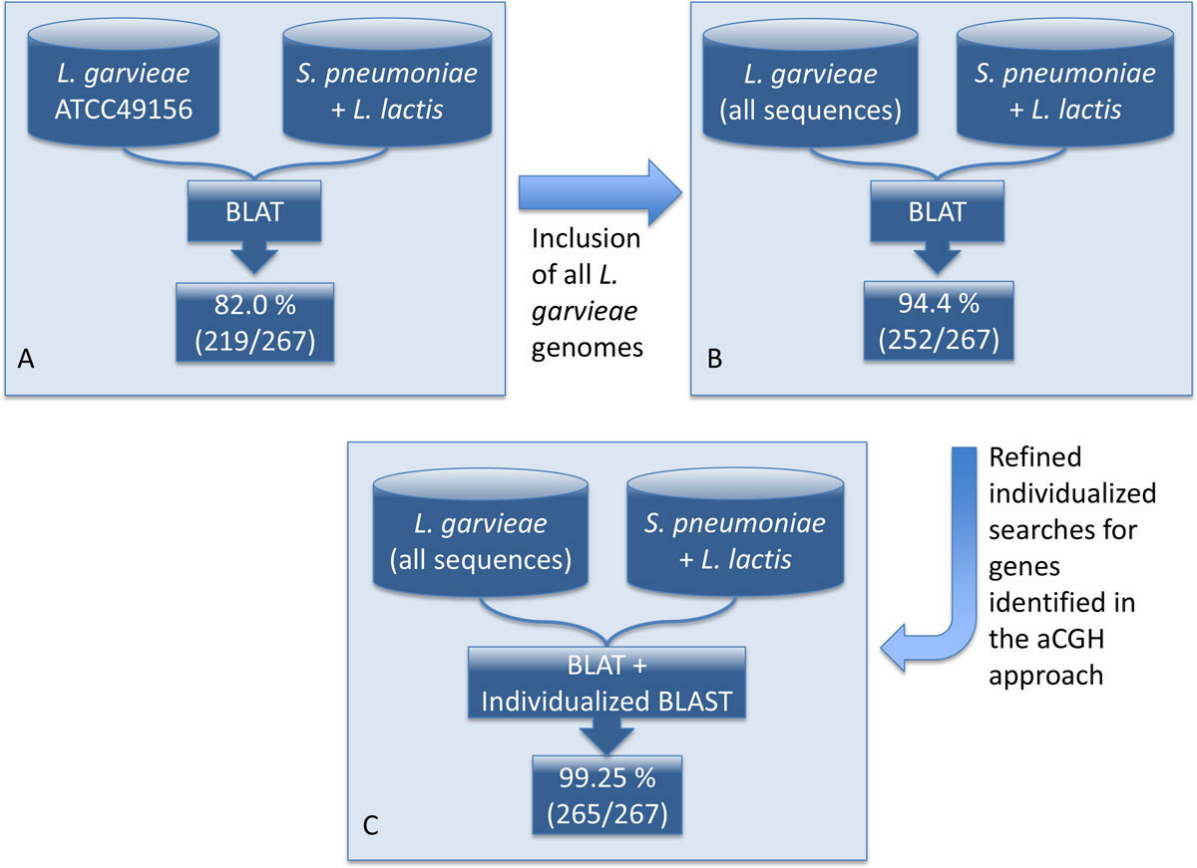
**Schema of the strategy followed for the validation of the aCGH after the release of the genome sequences for several *L. garvieae *strains**. The figure shows as well the accumulated validated genes obtained in each of the steps.

This validation analysis was carried out comparing the probes sequences against the currently considered reference genome (*L. garvieae *ATCC49156, Genbank: http://www.ncbi.nlm.nih.gov/nuccore/NC_015930.1) using BLAT (Figure 4). This analysis showed that up to the 82.0% of the identified genes (219) in the aCGH experiment are also present on this reference genome, and 18.0% (48) are missing due to either being genes strain specific or false positive calls from the methodology designed for the comparative genomics experiment. For further investigations on this set of missing genes, a second step consisted in the repetition of the previous BLAT sequence analysis in an extended set of sequences built up with all available sequences in Refseq and the whole genome shotgun contigs sequences from the NCBI for *L. garvieae*. This new analysis increased the sensitivity and the number of identified sequences up to 94.4% (252 out of the 267 proposed conserved genes).

The remaining 15 genes that could not be identified during the first two steps using the standard BLAT searches were analysed individually. As was expected from the phylogenetic relationship of the reference genomes used for the aCGH analyses, a majority (11) of these 15 genes were identified in the phylogentically more distant *S. pneumoniae *TIGR4 while only four belonged to the closer *L. lactis *subsp. *lactis *IL1403. Of these four, gene pi322 identified in *L. lactis *was removed for further analyses because it is a prophage. The individualized of these remaining 14 sequences was carried out using BLAST searches using NCBI's BLAST BLASTn algorithm (word size = 11). This analysis allowed the detection of 13 out of the 14 genes in one or more *L. garvieae *draft genome sequences. A special case is the gene SP0034: this gene is annotated as a hypothetical protein and it is the only one from the 267 identified genes by the aCGH that lacks supporting evidence based on current sequence information and comparison analyses based on searches either using the complete gene sequence or the microarray probe sequence. In the case of whole gene sequence analysis it is possible to identify a weak similarity based on a 41 bases alignment with 80% of identities whereas the probe sequence analysed shows only a 18 bases alignment with only three of the available genomes.

The results of these analyses, where an increase in the number of genomes included in the comparisons leads to an increased number of validated genes, show that there is an important variability in the genomic content of this bacteria depending on the strain analysed.

### Comparative genomics using whole genome

The availability of genome sequences have made possible the comparative study of different strains of *L. garvieae *at genomic level aiming to understand the pathogenicity of this organism and identifying candidate virulence genes [36]. Due to the fact that only two out of the eleven genomes are properly annotated, these approaches require an annotation step in order to proceed with further comparisons. Such experiments allowed identifying a core set of 1341 genes of *L. garvieae *[37]. From these studies the authors also were able to identify 16 potential virulence factors.

### Gene expression analyses

The annotation of the genome allowed for the design of gene expression arrays where short probes were designed with the aim to identify each gene from two different strain of *L. garvieae *(*L. garvieae *21881 *and L garvieae *8831). The design of the probes required making them unique for each of the genes, avoiding any similarity with other regions of the genome to prevent cross hybridization during the experiments.

Similarly to the aCGH experiments, gene expression arrays required the management of images and their analysis and transformation into numerical values for each of the 4500 probes contained on each microarray. After arrays have been quantified, the results are analysed statistically to compare the expression between two or more physiological conditions. For *L. garvieae *the only gene expression analyses available to the date was carried out comparing two different clinical strains (one isolated from trout epidemics, strain 8831, and another one isolated from a human septicemia, strain 21881) at two different temperatures [38].

The management of these analyses needed not only the annotation of the genomes required for the design of the probes, but also a deeper manual annotation combined with pathways analyses and analysis of operons for a proper interpretation of the results. This information required accessing different databases such as NCBI COGs [39], KEGG [40] and literature, among others. Another important characteristic associated with the development of almost any high throughput laboratory technique is the need to annotate the samples and all the processes carried out during the experiments in order to be able to provide them in a manner compliant with the data sharing standards, such as MIAME [41] for microarray experiments.

A key aspect related with gene expression analyses is the annotation of samples and processes carried out that are required by MIAME standard. This annotation includes information related with a variety of data types, such as concentration of different reagents, photo-spectrometry data related with efficiency in labeling processes and other experimental parameters, as well as a curated annotation of the probes on the microarray. This information was managed using a "in-house" built MIAME compliant system [42] that facilitated data analyses, results interpretation and data export to public repositories.

Microarrays transcriptomic measurements were validated using a different technique, qPCR, representing an additional data source and data type that had to be integrated into these results.

### Other data types and analyses

Despite the increasing importance of the high throughput molecular techniques in the microbiological laboratories, the low throughput molecular approaches and classical techniques and their advances are still a very valuable source of information. These techniques and methodologies represent a diverse data type source (for example gel images or bacterial growth rates) and volumes due to the multiplexing of some of these techniques. An example of these other data sources would be confocal microscopy. This technique has been used in the analyses of *L. garvieae *to study whether the bacterium is able to enter the cells or it stays out of them. During these studies, series of dozens of high-resolution images are captured representing different layers. These series of images, should be generated for each of the multiple fluorescent dyes, used to identify the bacterium and the cells, and must be replicated in order to apply statistical analyses on them. Therefore, the generation and management of these images represent a challenge for the laboratories due to the data volumes that could be generated during these experiments.

## Conclusion

In recent years, advances in the experimental techniques have revolutionized the way of working in the laboratories and have changed in many ways their informatics requirements due to an overwhelming amount and variety of data. Microarrays and MPS techniques represent data challenging techniques for microbiologists. The extension in the use of these techniques is not only forcing the laboratories to deal with them and adapt to them but also are expanding enormously the amounts of information available in public repositories. Therefore it has pushed the concept of "Big Data" into this domain and challenged the requirements for sound management and analysis of data processing techniques in the laboratories. The "Big Data" concepts of high volume, veracity and variety are nowadays part of the research in microbiology associated with the traditional use of multiple methods in the laboratory combined with the genomic high-throughput techniques.

A consequence of these changes is that they are enabling a change in the research paradigms moving from a hypothesis-driven to a data-driven one, facilitating as well pure "in silico" research based on the data available.

Biological interpretation of the experiments requires not only the analysis and integration of multiple data types generated as a results of those experiments but also an increasingly amount of data that available in public repositories. These data can be used and in many cases are necessary to perform in "*in silico*" experiments to generate interpretation frameworks to understand and correctly interpret bench experiments. The previously described framework for the interpretation of aCGH experiments and the subsequent validation of the similarity threshold are examples of this use of information stored in massive data repositories. The effect in the amount of data used for the validation of the aCGH results shows the importance of dealing with increasing amounts of data and the relevance of "Big Data" in microbiology.

*L. garvieae *is a good example of how the diversity of data sources can be combined and analysed together for a better knowledge of the genetic and biology of poorly understood microorganisms (Table 1). A comprehensive characterization and understanding of the biology of a pathogenic organism requires the support of bioinformatics for data integration and analyses due to the amounts of diverse data generated through variety of experiments in the form of established pipelines, as well as their modification or the development of new ones. The use of these methods improves our understanding of the biology of this microorganism and provides insights about its pathogenicity and the associated mechanisms of virulence involved in *L. garvieae *infections.

**Table 1:** Example of data types and sizes involved in the analyses of *L. garvieae*

Data source	Size or number of elements	Data type
Genbank - DNA database (April2014)	~ 1.8e8 sequences ~ 1.7e11 nucleotides	Sequence Nucleotide

WGS (Whole Genome Surveys) Database (April 2014)	~ 1.4e8 sequences ~ 6.2e11 nucleotides	Sequence Nucleotide

Gene Expression Microarray (x array)	~ 5.0e3 sequences ~ 3.0e4 nucleotides 200 Mb ~ 1.4e4 Spots	Sequence Nucleotide Image Numeric

aCGH Microarray (x array)	~ 2.5e3 sequences ~ 1.0e6 nucleotides 20 Mb ~ 3.0e3 spots	Sequence Nucleotide Image Numeric

Immunohistochemistry	~ 1.5 Gb	Image

qPCR (x gene)	2 files	Numeric

KEGG pathways	460	Pathways

Sequencing results (x genome)	~ 4.7e5 sequences ~ 1.6e8 nucleotides	Sequence Nucleotide

Electrophoresis Gels (x image)	2 Mb	Image

## List of abbreviations

aCGH: Array based Comparative Genomics Hybridization

MPS: Massive Parallel Sequencing

qPCR: Quantitative Polymerase Chain Reaction

## Competing interests

The authors declare that they have no competing interests

## Authors' contributions

GHL-C carried out the "in silico" analyses, designed the "in silico" analyses, participated in the experimental analyses, and drafted the manuscript. MA-U carried out the experimental analyses, participated in the "in silico" analyses and design, interpretation of the experimental analyses and drafted the manuscript. MMB Designed the experimental analyses, participated in the interpretation of the experimental analyses and collaborated in drafting the manuscript. AG participated in the experimental design participated in the interpretation of the experimental analyses and collaborated in drafting the manuscript. MTC participated in the interpretation of the experimental analyses. VLA participated in the "*in silico*" analyses, and collaborated in their design. FMS participated in the "in silico" analyses design and collaborated in drafting the manuscript. JFFG participated in the design of the experimental analyses, participated in the interpretation of the experimental analyses and collaborated in drafting the manuscript.

## Funding

This research was supported by grants AGL2009-12447 and AGL2012-35419 from the Spanish Ministry of Economy and Competiveness. The funders had no role in study design, data collection and analysis or preparation of the manuscript.

## Declarations Section

The publications costs of this paper were funded by the Health and Biomedical Informatics Centre of The University of Melbourne.

This article has been published as part of *Health Information Science and Systems *Volume 3 Supplement 1, 2015: Proceedings of the Health Informatics Society of Australia Big Data Conference (HISA 2013). The full contents of the supplement are available online at http://www.hissjournal.com/supplements/3/S1/
